# L-Arginine/NO Pathway Is Altered in Children with Haemolytic-Uraemic Syndrome (HUS)

**DOI:** 10.1155/2014/203512

**Published:** 2014-03-17

**Authors:** Nele Kirsten Kanzelmeyer, Lars Pape, Kristine Chobanyan-Jürgens, Dimitrios Tsikas, Hans Hartmann, Anne-Jule Fuchs, Bernhard Vaske, Anibh Martin Das, Marion Haubitz, Jens Jordan, Thomas Lücke

**Affiliations:** ^1^Department of Paediatric Kidney, Liver and Metabolic Diseases, Hannover Medical School, Carl-Neuberg-Straße 1, 30625 Hannover, Germany; ^2^Institute of Clinical Pharmacology, Hannover Medical School, Carl-Neuberg-Straße 1, 30625 Hannover, Germany; ^3^Department of Paediatrics, University of Mainz, Langenbeckstraße 1, 55131 Mainz, Germany; ^4^Institute of Biometry, Hannover Medical School, Carl-Neuberg-Straße 1, 30625 Hannover, Germany; ^5^Department of Medicine III, Klinikum Fulda, Pacelliallee 4, 36043 Fulda, Germany; ^6^Department of Neuropaediatrics, University Children's Hospital, Ruhr University Bochum, Gudrunstraße 56, 44791 Bochum, Germany

## Abstract

The haemolytic uraemic syndrome (HUS) is the most frequent cause of acute renal failure in childhood. We investigated L-arginine/NO pathway in 12 children with typical HUS and 12 age-matched healthy control subjects. Nitrite and nitrate, the major NO metabolites in plasma and urine, asymmetric dimethylarginine (ADMA) in plasma and urine, and dimethylamine (DMA) in urine were determined by GC-MS and GC-MS/MS techniques. Urinary measurements were corrected for creatinine excretion. Plasma nitrate was significantly higher in HUS patients compared to healthy controls (*P* = 0.021), whereas urine nitrate was borderline lower in HUS patients compared to healthy controls (*P* = 0.24). ADMA plasma concentrations were insignificantly lower, but urine ADMA levels were significantly lower in the HUS patients (*P* = 0.019). Urinary DMA was not significantly elevated. In HUS patients, nitrate (*R* = 0.91) but not nitrite, L-arginine, or ADMA concentrations in plasma correlated with free haemoglobin concentration. Our results suggest that both NO production and ADMA synthesis are decreased in children with typical HUS. We hypothesize that in the circulation of children with HUS a vicious circle between the L-arginine/NO pathway and free haemoglobin-mediated oxidative stress exists. Disruption of this vicious circle by drugs that release NO and/or sulphydryl groups-containing drugs may offer new therapeutic options in HUS.

## 1. Introduction

Haemolytic-uraemic syndrome (HUS) is the most frequent cause of acute renal failure in childhood [1]. HUS is defined by the triad of haemolytic anaemia, acute renal failure, and thrombocytopenia. HUS in childhood is predominantly induced by an infection with verocytotoxin- (Shiga-like toxin-) producing bacteria, typically* Escherichia coli*. HUS primarily occurs in children one to 10 years of age with an average annual incidence of one to three cases per 100,000 children and a survival rate of nearly 95% [2]. Beside the outbreak of Shiga-toxin producing* E. coli* O104:H4 in 2011 [3], in Austria and Germany, the incidence is 0.4 : 100,000 and 0.7 : 100,000, respectively [4]. The disease begins after an incubation period of 4 to 7 days with abrupt onset of bloody diarrhoea and abdominal pain. Two to ten days later, microangiopathy, haemolytic anaemia, thrombocytopenia, and acute renal failure develop. Specific pathological findings in HUS patients with acute renal failure are glomerular microthrombi [5, 6]. HUS microangiopathy can involve almost any organ, but damage to kidneys and central nervous system cause the most severe clinical problems [7]. Despite the often dramatic clinical presentation, the overall outcome of childhood HUS is relatively good. Mortality is less than 5%, and 75% of patients show complete remissions [8]. Relapses are rare.

Shiga-like toxin binds to the glycosphingolipid globotriaosylceramide [9], thus interfering with protein synthesis in endothelial cells. Disordered von Willebrand factor, platelet activation via platelet-activating factor, interleukins, and nitric oxide (NO) may also contribute to the disorder [7]. NO has multiple functions including regulation of vascular tone, neurotransmission, and inhibition of platelet aggregation and leukocyte adhesion [10]. Antiproliferative and antiatherosclerotic effects have also been ascribed to NO [11].

NO is a short-lived free radical which is produced in all types of cells including endothelial cells. In vivo, NO is rapidly oxidized to nitrate and nitrite, which circulate in blood and are excreted in the urine [12]. Under certain conditions circulating nitrite and nitrate and excretory nitrate are suitable indicators and measures of NO synthesis [13]. Thus, nitrate in urine is considered a useful measure of whole body NO synthesis, whereas circulating nitrite rather reflects endothelium-dependent NO synthesis. NO is generated from the amino acid L-arginine by NO synthases (NOS) isoforms, including endothelial NOS (eNOS), neuronal NOS (nNOS), and inducible NOS (iNOS). These enzymes oxidize the imino group of the terminal guanidine group of L-arginine to NO, with L-citrulline being the second reaction product [14].

NOS activity is regulated by endogenous inhibitors, with asymmetric dimethylarginine (ADMA) being the most important [15]. ADMA is generated by methylation of protein-associated L-arginine catalyzed by N-methyl protein transferases followed by regular proteolysis [16]. Unchanged ADMA is excreted by the kidneys, but the greatest part of endogenously produced ADMA (about 90%) is excreted in the urine as dimethylamine (DMA) after hydrolysis by the enzyme dimethylarginine dimethylaminohydrolase (DDAH) [15], predominantly in kidney and liver. High circulating ADMA levels were found in many diseases including chronic renal failure [17].

In adult patients suffering from different microangiopathic diseases NO synthesis seems to be increased [18]. Yet, in childhood data on the L-arginine/NO pathway is rare. We recently reported reference data for the different members of the NO family in childhood [19]. We also studied these parameters in children suffering from renal diseases [20]. As microangiopathy also occurs in HUS patients, we were interested to see if the L-arginine/NO pathway is altered in children with HUS. It is worth mentioning that in the acute phase of HUS, erythrocytes are exposed to elevated oxidative stress that could contribute to haemolysis directly through oxidative damage and/or decreasing membrane fluidity [21]. More recently, it was demonstrated that during the acute phase of HUS in humans oxidative stress is elevated when measured as lipid peroxidation products in plasma [22]. In mice, Shiga toxin 2 was shown to increase oxidative stress and antioxidants such as* N*-acetylcysteine were found to ameliorate Shiga toxin-induced oxidative stress measured as malondialdehyde and to decrease renal damage [23]. In a patient, a 9-year-old girl, suffering from HUS after transplantation transdermal application of isosorbide dinitrate (ISDN), a NO-releasing drug was found to be very effective in ameliorating haemolysis and in increasing the number of platelets [24]. This interesting finding indicates that NO may play a protective role in HUS. In consideration of the potential involvement of the L-arginine/NO pathway in children suffering from typical HUS, we wanted to characterize quantitatively and comprehensibly the status of HUS by measuring several members of the L-arginine/NO pathway in plasma and urine. Healthy children served as the control group in the present study. As nitrite in human urine may potentially indicate nitrative stress [25], we quantitated urinary nitrite in addition to urinary nitrate which is a measure of whole-body NO synthesis [13].

## 2. Materials and Methods

Twelve children with typical HUS (5 girls, 7 boys; mean age 3.6 ± 3.5 years) and twelve age-matched healthy controls were included. The clinical characteristics of the patients are summarized in Table 1. Patients were treated symptomatically with diuretics and dialysis. If arterial hypertension was present they received antihypertensive drugs (Table 1). Patients were discharged about two weeks after the end of dialysis which was performed for 5 to 30 days (Table 1). Blood and, where possible, urine were taken at the first or second day of inpatient stay. The study was approved by the Ethics Committee of the Hannover Medical School and written consent was obtained from the parents.

ADMA in plasma and urine and L-arginine in plasma were determined by GC-MS/MS and GC-MS, respectively, as described elsewhere [26]. Nitrite and nitrate in plasma and urine were determined simultaneously by GC-MS as described previously [27]. Urinary creatinine was determined by GC-MS [28]. DMA in urine was determined by GC-MS as described recently [29]. Urinary excretion of the analytes was corrected for creatinine excretion and data are presented as *μ*mol of the analyte per mmol of creatinine.

As a measure of endothelial damage, circulating endothelial cells were detected in blood at the time of admission as described by us elsewhere [30].

Data from patients and healthy controls were compared using the Mann-Whitney test (SPSS, version 16). Data are presented as mean ± SD. Values of *P* < 0.05 were considered significant.

## 3. Results

Upon admission, the number of circulating endothelial cells in the HUS childrens' blood was 44 per mL (median, range 0 to 800 per mL), indicating endothelial damage [30]. At time of discharge, the number of circulating endothelial cells decreased to 24 per mL (median, range 0 to 180 per mL).

**Figure 1:**
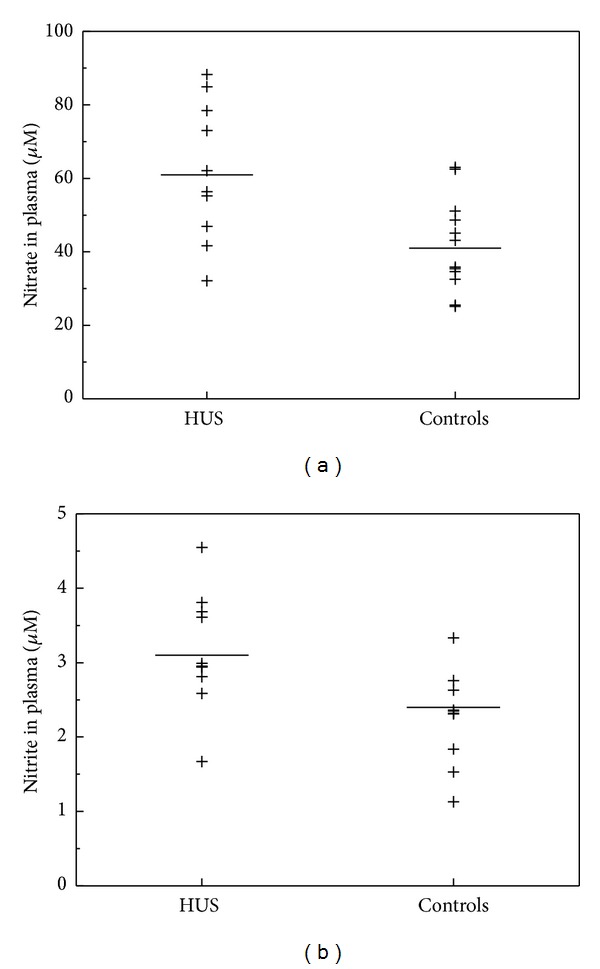
Plasma concentrations of nitrate (a) and nitrite (b) in children with haemolytic-uraemic syndrome (HUS, *n* = 12) and in healthy children (controls, *m* = 12). Horizontal bars indicate the mean values. Nitrate (*P* = 0.021) and nitrite concentrations were significantly (*P* = 0.017) higher in HUS as compared with controls.

**Figure 2:**
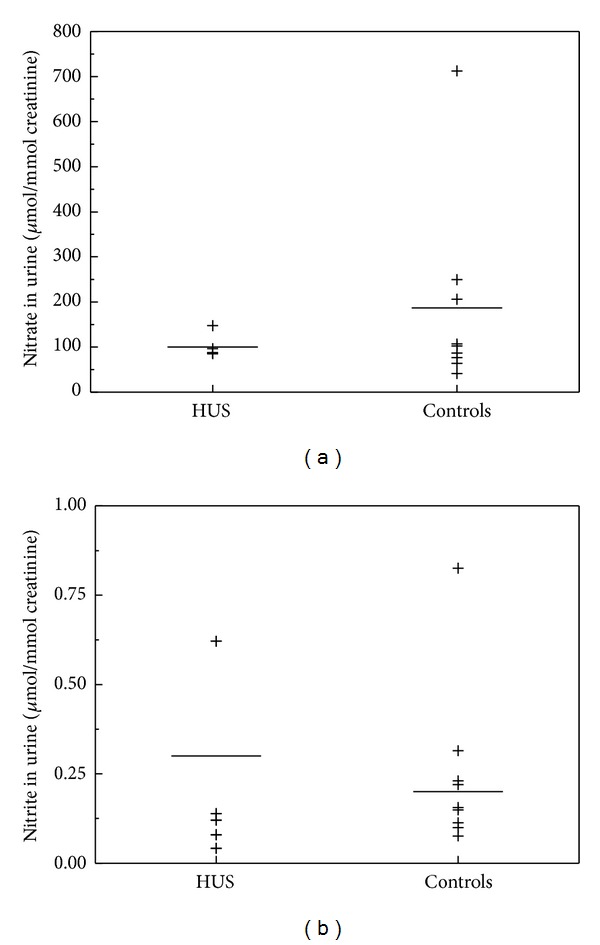
Urinary excretion of nitrate (a) and nitrite (b) in children with haemolytic-uraemic syndrome (HUS, *n*) and in healthy children (controls, *m*). Horizontal bars indicate the mean values. Nitrate and nitrite excretion rates did not differ significantly between HUS and control (*P* = 0.24 and *P* = 0.58, resp.; each *n* = 5, *m* = 9).

In children with HUS, plasma nitrate (61.9 ± 18.9 versus 41.5 ± 13.1 *μ*M, *P* = 0.021) and plasma nitrite (3.1 ± 0.8 versus 2.4 ± 1.1 *μ*M, *P* = 0.017) were significantly increased, as compared to the healthy controls (Figure 1). Excretion rates of nitrate were insignificantly lower in the HUS patients as compared to the healthy controls (100 ± 27 versus 187 ± 207 *μ*mol/mmol, *P* = 0.24) (Figure 2(a)). Urinary nitrite did not differ between HUS and healthy controls (0.3 ± 0.2 versus 0.2 ± 0.2 *μ*mol/mmol, *P* = 0.58) (Figure 2(b)).

**Figure 3:**
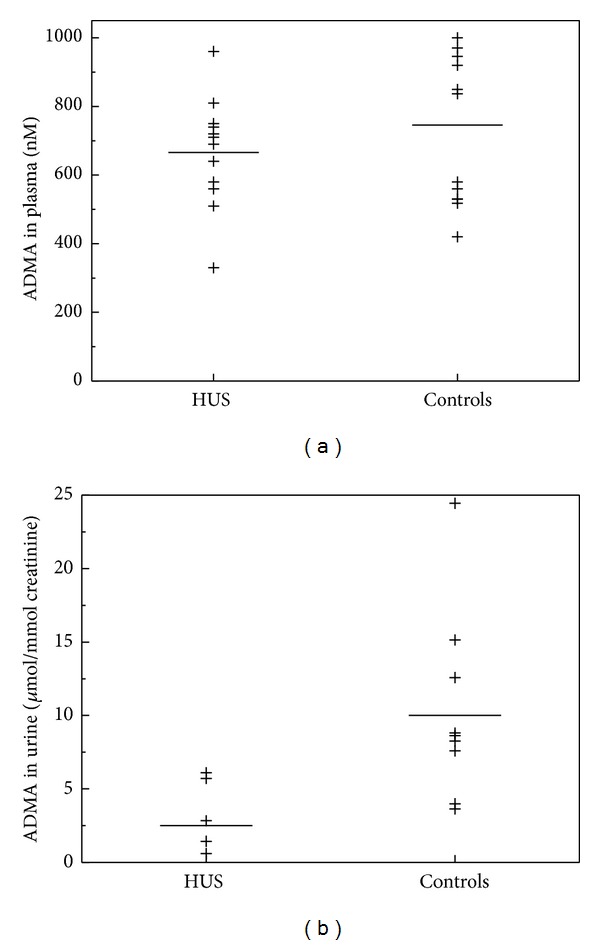
Plasma concentrations (a) and urine excretion rates (b) of ADMA in children with haemolytic-uraemic syndrome (HUS, *n*) and in healthy children (controls, *m*). Horizontal bars indicate the mean values. Plasma ADMA concentrations were insignificantly lower in HUS as compared with controls (*P* = 0.32; *n* = 12, *m* = 12). Urinary excretion of ADMA was significantly lower in HUS as compared with controls (*P* = 0.019; *n* = 5, *m* = 9).

**Figure 4:**
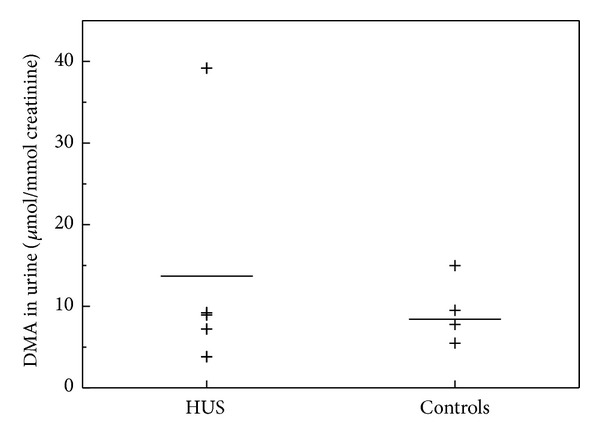
Urinary excretion rate of DMA in children with haemolytic-uraemic syndrome (HUS; *n* = 4) and in healthy children (controls, *m* = 5). Horizontal bars indicate the mean values. DMA levels were insignificantly higher in HUS as compared with controls (*P* = 1.0).

**Figure 5:**
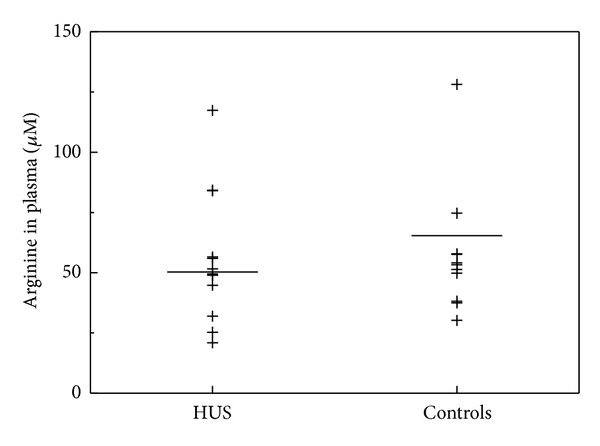
Plasma concentrations of arginine in children with haemolytic-uraemic syndrome (HUS; *n*) and in healthy age-matched children (controls, *m*). Horizontal bars indicate the mean values. Arginine concentrations did not differ between patients and healthy controls (*P* = 0.48; *n* = 12, *m* = 12).

Plasma ADMA concentrations were insignificantly lower in the HUS children as compared to the healthy children (666 ± 160 versus 746 ± 208 nM, *P* = 0.32) (Figure 3(a)). However, renal excretion rate of ADMA was significantly lower in patients with HUS compared to healthy controls (3.3 ± 2.5, *n* = 5, versus 10.1 ± 6.5, *n* = 9) *μ*mol/mmol, *P* = 0.019) (Figure 3(b)). Excretion rate of DMA in urine was insignificantly higher in patients with HUS, that is, 13.7 ± 14.4 (*n* = 4) in comparison to 8.4 ± 5.6 (*n* = 5) *μ*mol/mmol in healthy children (*P* = 1.0) (Figure 4). Plasma L-arginine plasma levels were not statistically different between the two groups (Figure 5; *P* = 0.48).

**Figure 6:**
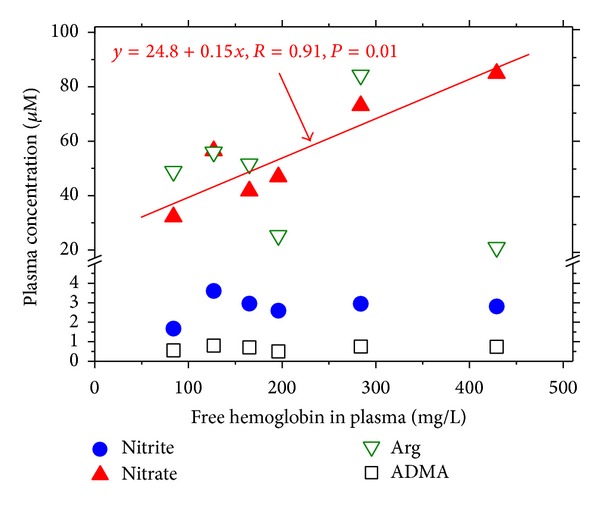
Relationship between nitrite, nitrate, L-arginine, or ADMA in plasma and free hemoglobin in plasma of the children with haemolytic-uraemic syndrome investigated in the present study.

In the HUS patients, there was a close positive correlation (*R* = 0.91, *P* = 0.01) between plasma nitrate concentration (*y*) and plasma free haemoglobin concentration (*x*) with the regression equation *y* = 25 + 0.15*x*; by contrast, plasma nitrite, L-arginine, and ADMA concentration did not correlate with plasma free haemoglobin concentration (Figure 6).

## 4. Discussion

The L-arginine/NO pathway plays an important role in renal failure, infection, and microangiopathy [18, 31, 32]. In adult patients with microangiopathy endogenous NO production seems to be elevated [18]. In the present study we investigated the status of the L-arginine/NO pathway in children with typical haemolytic uraemic syndrome (HUS), which is associated with haemolytic anaemia, acute renal failure, and thrombocytopenia.

The number of free circulating endothelial cells in children with HUS at the time of admission was much higher than that commonly found in healthy individuals [30]. These expected findings suggest that endothelial damage occurred in the HUS patients investigated in the present study. At time of discharge, the number of circulating endothelial cells decreased to normal levels, indicating improvement of endothelial dysfunction.

**Figure 7:**
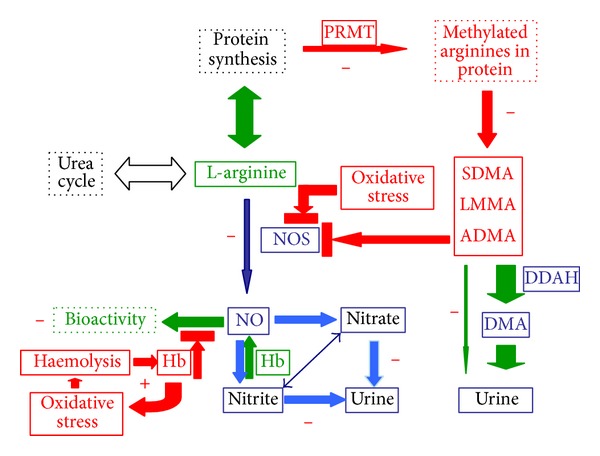
Proposal of the status of the L-arginine/NO pathway in the haemolytic-uraemic syndrome (HUS) in childhood. The enzyme NOS converts L-arginine into L-citrulline (not shown) and NO which plays multiple physiological roles. NO autooxidizes to nitrite. In red blood cells, NO is oxidized to nitrate. Nitrite and nitrate are excreted in the urine. By far the major part of L-arginine participates in the urea cycle and is utilized in protein synthesis. PRMT methylate L-arginine in proteins and methylated proteins are hydrolyzed to the soluble L-arginine derivates SDMA, LMMA, and ADMA. LMMA and ADMA act as inhibitors of NOS, thus controlling NO synthesis. ADMA is hydrolyzed by DDAH to L-citrulline (not shown) and DMA which is excreted in the urine. The status of the L-arginine/NO pathway can be described satisfactorily by measuring nitrite, nitrate, ADMA, L-arginine, and DMA in plasma or serum and urine. In HUS, NO and ADMA biosynthesis is diminished, whereas DDAH activity is not altered. Oxidative stress is elevated, haemolysis is increased, which releases Hb that in turn elevates oxidative stress, thus finally establishing a vicious circle. Minus means diminished; plus means increased. Abbreviations used in this Figure: ADMA: asymmetric dimethylarginine; DDAH: dimethylarginine dimethylaminohydrolase; DMA: dimethylamine; LMMA: monomethyl arginine; NOS: nitric oxide synthase; PRMT: protein arginine methyl transferase; SDMA: symmetric dimethylarginine.

To study the L-arginine/NO pathway we obtained blood and urine samples at the first or second day of inpatient stay. In the plasma samples of our patients with typical HUS we observed significantly increased concentrations of nitrate and nitrite compared to healthy children. In former studies of our groups, nitrate and nitrite concentrations were not elevated in children with syndromic [33] and nonsyndromic focal-segmental-glomerulosclerosis (FSGS) or in children with non-FSGS renal diseases [20]. Taken all together, impaired renal function in HUS patients is likely to be a reason for accumulating plasma nitrite and nitrate concentrations rather than an enhanced NO synthesis (Figure 7). In the children of the present study, we did not measure cGMP, the second messenger of NO, in plasma or urine samples. In forthcoming studies, measurement of cGMP in plasma of HUS children could provide additional, valuable information about NO biosynthesis/bioavailability in this syndrome, although circulating or urinary cGMP and NO synthesis or bioactivity are not dependable correlates.

Unfortunately, urine samples were not available from all HUS children and the power of the biochemical parameters measured in urine samples is considered rather low. While plasma ADMA concentrations were not increased in the HUS patients, urinary excretion rate of ADMA in HUS patients was almost threefold lower than in healthy children. Analogous to urinary nitrate, excretion of unchanged ADMA in the urine seems to be impaired most likely due to renal failure. These results and the observation that DMA excretion rates were similar in the HUS patients and the healthy controls suggest that ADMA synthesis rate is decreased in typical HUS, presumably due to impaired activity of* N*-methyl protein transferases and/or impaired activity of proteolytic enzymes in this syndrome (Figure 7). On the other hand, our results suggest that DDAH activity is not altered in HUS. Deficiency of L-arginine, the common precursor of NO and ADMA, is unlikely to explain the reduction in ADMA synthesis in the HUS patients, as plasma L-arginine concentrations were similar in patients and in control subjects.

Elevated NO production has been observed in a mouse model of Shiga toxin 2-induced HUS [34]. Shao et al. [35] suggested eNOS upregulation in a rat model for thrombotic microangiopathy and that elevated endothelial NO synthesis could be an important protective mechanism in thrombotic microangiopathy [35]. NO production by neutrophil leucocytes following stimulation with Shiga toxin has been found to be age-dependent with lower production in infants; the authors speculate that this may be related to the higher incidence in infants [36]. As nitrite and nitrate in plasma and/or in urine do not reflect the activity of a particular NOS isoform or organ, our results do not allow the drawing of any conclusions regarding eNOS contribution to NO in the HUS patients of healthy children. Therefore, in our HUS patients we cannot exclude elevation in expression and activity of iNOS which generates NO for purpose of antimicrobial defense, but we consider it rather very moderate.

The intact erythrocyte plays an important role both in NO inactivation through oxyhaemoglobin and in storage and transport of NO bioactivity within the circulation [37–42]. Therefore, it is likely that haemoglobin species inside as well as outside of the erythrocyte, that is, free haemoglobin, are of particular importance for NO-related biological actions in HUS patients, in a way independent of the endothelial function. Free haemoglobin in plasma may act both as a trap for NO and as a producer of NO from inorganic nitrite, the autoxidation product of NO (Figure 7). Hypoxia is not atypical for HUS. Disturbed microcirculation in HUS caused by thrombotic microangiopathy may lead to hypoxia which facilitates nitrite reduction to NO by free haemoglobin. In this situation, additionally produced NO from nitrite may be meaningful and beneficial by acting as inhibitor of aggregation as well as a vasodilatator. In addition, haemoglobin may produce vasoactive substances such as prostaglandins and thromboxane, but it may also induce oxidative stress [43]. In the present study, we found a positive correlation between nitrate and free haemoglobin in plasma. This observation together with the almost uniform distribution of nitrite and nitrate in blood cells including erythrocytes [44, 45] may argue for oxidation of NO to nitrate by free haemoglobin outside of the erythrocyte rather than for nitrate release by damaged erythrocytes. It is worthy of mention that haemolysis in our HUS patients was not higher than about 0.2%. However, because free haemoglobin in the plasma is several times more reactive against NO than haemoglobin inside of the erythrocytes, it is likely that a large fraction of NO has been oxidized to nitrate by free haemoglobin in the plasma of our HUS patients. In HUS, like in sickle cell anemia [41], haemolysis seems to be associated with harmful rather than with beneficial effects. Cell-free haemoglobin released by haemolysis may oxidize NO to biologically inactive nitrate as mentioned above. In addition, free haemoglobin may also inactivate NO by enhancing oxidative stress, for instance, by producing reactive oxygen species (ROS) such as superoxide radical anions and hydrogen peroxide. Such ROS are highly reactive and may therefore oxidize NO to peroxynitrite. This is a strong oxidant on its own and will finally decompose to nitrite, nitrate and dioxygen. It is likely that these deleterious effects of haemoglobin are not exerted in a stoichiometric manner [43].

Based on our results suggesting a decreased synthesis rate of endogenous NO in HUS, one may speculate that basal NO synthesis is insufficient to ameliorate thrombotic microangiopathy in this syndrome and that exogenous NO in pharmacological doses may therefore be required. In a 9-year-old girl suffering from HUS following bone marrow transplantation at the age of 8 months, 20 mg/day transdermal ISDN for 9 weeks ameliorated haemolysis while increasing platelet counts. The girl did not experience side effects or the disease did not recur after cessation of ISDN treatment [24]. Interestingly, many of the clinical characteristics of this girl were comparable to those of our HUS patients. Unfortunately, no data had been reported about the L-arginine/NO pathway in this girl. It is worth mentioning that ISDN was found not to increase oxidative stress in healthy young subjects when applied at a therapeutically relevant dose of 30 mg thrice a day [46]. In contrast, at this dose ISDN appeared to decrease basal nitrative stress one day after administration when measured both as soluble 3-nitro-tyrosine and 3-nitro-tyrosine-albumin, though not statistically significant [46], which are potential biomarkers for NO-dependent oxidative stress in humans [47]. Due to the limited availability of urine samples and the artefactual contribution of haemolysis to biomarkers of oxidative stress such as malondialdehyde (MDA) and the F_2_-isoprostane 15(*S*)-8-*iso*-PGF_2*α*_ [43], but not to ADMA and other members of the L-arginine/NO pathway [48], we abandoned the analysis of MDA and 15(*S*)-8-*iso*-PGF_2*α*_ in the plasma and urine samples of our study.

In adults CAD patients we found that low urinary ADMA excretion rates are associated with impaired cardiac function and predict cardiovascular as well as all-cause mortality [49]. In the present study we measured in HUS children for the first time lower ADMA excretion rates than in healthy children. The potential clinical value of urinary ADMA for the diagnosis of CAD or cardiac dysfunction in adults and for the diagnosis of HUS in childhood warrants further studies.

In summary, the L-arginine/NO pathway is altered in childhood HUS. NO synthesis seems to be diminished despite a decreased synthesis of ADMA, an endogenous inhibitor of NO synthesis from L-arginine, compared to healthy age-matched children. Free haemoglobin is likely to play an important role in the metabolism and biological activity of NO, including endothelium-derived NO, and to induce oxidative stress, for instance, lipid peroxidation in blood, thus damaging the erythrocyte membrane and causing haemolysis. Further studies are required to delineate the relative contribution of damaged erythrocytes and damaged endothelium to HUS. Application of drugs with the potential to release NO, such as the organic nitrate ISDN, could be effective as a therapeutic means in the treatment of HUS. The underlying mechanism leading to impaired NO synthesis in HUS is unclear and demands further elucidation. The potential beneficial effect of organic nitrates on haemolysis and platelets and the underlying mechanism(s) warrant further investigations. It appears that oxidative stress is elevated in HUS while NO synthesis is decreased. Supplementation of children suffering from HUS with NO-releasing drugs or drugs that increase NO synthesis and/or bioavailability and that decrease oxidative stress, such as* N*-acetylcysteine (NAC) or its more lipophilic and cell membrane-permeable* N*-acetylcysteine ethyl ester (NACET) [50], may be useful measures among others in the treatment of HUS.

## Figures and Tables

**Table 1:** Clinical characteristics of the haemolytic uraemic syndrome (HUS) patients on the 1st or 2nd day of admission and duration of peritoneal dialysis.

Patient number	Duration of peritoneal dialysis (days)	Neurological events	Hypertension	Serum creatinine (µM)	Serum urea (mM)	Urine albumin (g/L)	Total hemoglobin (g/dL)	Free hemoglobin (mg/L)	Lactate dehydrogenase (U/L)
1	0	No	No	108	15.4	3.7	6.6	429	2448
2	7	No	No	394	28.4	0.37	7.6	196	968
3	30	Ataxy	Yes	256	35.0	0.05	6.8	127	3789
4	5	No	No	458	41.9	0.41	5.9	165	2606
5	9	EEG alteration	No	132	20.4	Not measured	6.8	155	1462
6	24	No	Yes	209	21.0	0.89	5.7	Not measured	2611
7	30	Status epilepticus	No	304	26.3	0.24	4.8	Not measured	2593
8	10	No	No	50	7.8	4.45	6.9	84	3090
9	8	EEG alteration	No	528	39.4	Not measured	13.0	Not measured	2003
10	6	No	No	600	68.0	Not measured	8.2	Not measured	Not measured
11	20	No	Yes	373	19.8	3.4	6.0	Not measured	2662
12	14	EEG alteration	No	296	28.4	4.97	6.2	284	2379
